# Supplementary material for: Is It Time to Change Our Reference Curve for Femur Length? Using the Z-Score to Select the Best Chart in a Chinese Population
Source: PLoS One. 2016 Jul 26;11(7):e0159733. doi: 10.1371/journal.pone.0159733 (PMC4961440; doi:10.1371/journal.pone.0159733)
Supplement: S2 File — (PDF) [file pone.0159733.s002.pdf]

AMERICAN JOURNAL EXPERTS

EDITORIAL CERTIFICATE

This document certifies that the manuscript listed below was edited for proper English language, grammar, punctuation, spelling, and overall style by one or more of the highly qualified native English speaking editors at American Journal Experts.

Manuscript title:

Is it time to change our reference curve for femur length?——Using Z-score to select the best chart in Chinese population

Authors:

Boya Li, Huixia Yang,Yumei Wei, Chen Wang1, Rina Su1, Wenying Meng2, Yongqing Wang, Lixin Shang, Zhenyu Cai, Liping Ji6, Yunfeng Wang7, Ying Sun8, Jiaxiu Liu, Li Wei, Yufeng Sun, Xueying Zhang12, Tianxia Luo13, Haixia Chen, and Lijun Yu

Date Issued:

March 29, 2016

Certificate Verification Key:

D29D-67C9-1E8F-57AB-618D

This certificate may be verified at www.aje.com/certificate. This document certifies that the manuscript listed above was edited for proper English language, grammar, punctuation, spelling, and overall style by one or more of the highly qualified native English speaking editors at American Journal Experts. Neither the research content nor the authors' intentions were altered in any way during the editing process. Documents receiving this certification should be English-ready for publication; however, the author has the ability to accept or reject our suggestions and changes. To verify the final AJE edited version, please visit our verification page. If you have any questions or concerns about this edited document, please contact American Journal Experts at support@aje.com.

American Journal Experts provides a range of editing, translation and manuscript services for researchers and publishers around the world. Our top-quality PhD editors are all native English speakers from America's top universities. Our editors come from nearly every research field and possess the highest qualifications to edit research manuscripts written by non-native English speakers. For more information about our company, services and partner discounts, please visit www.aje.com.
