# Supplementary material for: Is It Time to Change Our Reference Curve for Femur Length? Using the Z-Score to Select the Best Chart in a Chinese Population
Source: PLoS One. 2016 Jul 26;11(7):e0159733. doi: 10.1371/journal.pone.0159733 (PMC4961440; doi:10.1371/journal.pone.0159733)
Supplement: S3 File — The data of this version was different from the publication one because the exclusion criteria had changed after the second revision. (DOC) [file pone.0159733.s003.doc]

## Is it time to change our reference curve for femur length? Using the Z-score to select the best chart in a Chinese population

Boya Li^1^, Huixia Yang^1^*, Yumei Wei^1^, Chen Wang^1^, Rina Su^1^, Wenying Meng^2^, Yongqing Wang^3^, Lixin Shang^4^,Zhenyu Cai^5^, Liping Ji^6^, Yunfeng Wang^7^, Ying Sun^8^, Jiaxiu Liu^9^, Li Wei^10^, Yufeng Sun^11^, Xueying Zhang^12^,Tianxia Luo^13^, Haixia Chen^14^, and Lijun Yu^15^

**Abstract:**

**Objective:** To use Z-scores to compare different charts of femur length (FL) applied to our population with the aim of identifying the most appropriate chart.

**Methods:** A retrospective study was conducted in Beijing. Fifteen hospitals in Beijing were chosen as clusters using a systemic cluster sampling method, in which 15,194 pregnant women delivered from June 20th to November 30th, 2013. The measurements of FL in the second and third trimester were recorded, as well as the last measurement obtained before delivery. Based on the inclusion and exclusion criteria, we identified FL measurements from 20,089 ultrasounds from 7,330 patients between 11 and 43 weeks gestation. The FL data were then transformed into Z-scores that were calculated using three series of reference equations obtained from three reports: Leung TN, Pang MW et al (2008); Chitty LS, Altman DG et al (1994); and Papageorghiou, AT et al (2014). Each Z-score distribution was presented as the mean and standard deviation (SD). Skewness and kurtosis and were compared with the standard normal distribution using the Kolmogorov-Smirnov test. The histogram of their distributions was superimposed on the non-skewed standard normal curve (mean=0, SD=1) to provide a direct visual impression. Finally, the sensitivity and specificity of each reference chart for identifying fetuses <5th or >95th percentile (based on the observed distribution of Z-scores) were calculated. The Youden index was also listed. A [scatter](http://dict.youdao.com/w/scatter/) [diagram](http://dict.youdao.com/w/diagram/) with the 5^th^, 50^th^, and 95^th^ percentile curves calculated from and superimposed on each reference chart was presented to provide a visual impression.

**Results:** The three Z-score distribution curves appeared to be normal, but none of them matched the expected standard normal distribution. In our study, the Papageorghiou reference curve provided the best results, with a sensitivity of 100% for identifying fetuses with measurements < 5^th^ and ＞ 95^th^ percentile, and specificities of 99.8% and 81.6%, respectively.

**Conclusions:** It is important to choose an appropriate reference curve when defining what is normal. The Papageorghiou reference curve for FL seems to be the best fit for our population. Perhaps it is time to change the reference curve for femur length.

**Introduction**

The widespread use of ultrasound allows the measurement of fetal biometry and the estimation of fetal growth, thus making is possible to identify abnormal fetal growth patterns antenatally.

Of all routine ultrasound measurements, femur length (FL) is unique. It is not only a parameter that can assess fetal size but can also alert clinicians to the possible presence of fetal chromosomal abnormalities, intrauterine growth restriction and fetal malformations, particularly skeletal dysplasia, when it is below the expected range (5^th^ percentile).

However, a short FL does not always indicate abnormal fetal growth. In our clinical practice, we have found that a number of fetuses with a “short femur length” were very healthy. This may, in part, be because Down’s syndrome screening has become a routine risk assessment for aneuploidy in China, and women with a high risk of aneuploidy are offered amniocentesis. Most women choose to terminate their pregnancy when an aneuploidy such as trisomy 21, 18 or 13 is diagnosed. Nevertheless, our popular fetal charts should also be reexamined, and the following questions, addressed: are these charts of high quality in terms of both design and [statistical](http://dict.youdao.com/w/statistical/) [method](http://dict.youdao.com/w/method/)ology? Are they applicable in a Chinese population?

In 2014, the Fetal Growth Longitudinal Study of the INTERGROWTH-21st Project, a multi-center, population-based longitudinal study, published their data and recommended international fetal growth standards for the clinical interpretation of routine ultrasound measurements and for comparisons across populations[1]. Because this study involved a large population and was of high quality, it raised the following question with respect to the older charts: is it time for a change?

As mentioned by McCarthy EA et al, “Inconsistent chart use and overestimation of fetal smallness can result in cynicism, confusion, and anxiety for pregnant women and their caregivers at all stages of pregnancy”[2]. In this article, we focused on FL, using Z-scores that integrate the measurement itself, the mean and the SD into a single value[3] to compare different charts of FL in our population to identify the most appropriate chart.

We did not include measurements of abdominal circumference, head circumference and estimated fetal weight in our analysis.

**Methods**:

**Study design and participants**

A retrospective study was conducted in Beijing. Fifteen hospitals in Beijing were chosen as clusters using a systemic cluster sampling method, in which 15,194 pregnant women delivered from June 20^th^ to November 30^th^, 2014. The questionnaire was designed to obtain information by interviewing all patients and reviewing their medical records. We had access to identifying information during and after data collection. These hospital units managed both low- and high-risk obstetric populations. More than 99.9% of the parturients were Chinese.

FL data from the second and third trimesters measured in views showing the entire diaphysis were recorded, as well as the last measurement before delivery. Thus, we excluded the possibility of overestimated measurements due to excessive performance of ultrasounds by physicians who may have had certain concerns about a pregnancy. In some cases, such as preterm labor, we were unable to obtain three FL recordings.

**Ethics Statement**

The study was reviewed and approved by the Institutional Review Board of the First Hospital, Peking University (Reference number: 2013[572]). All participants provided written informed consent, and the Ethics Committee approved the consent procedure.

**Exclusion criteria:**

Because most reference curves were developed based on “normal” pregnancies, we excluded women who were at high risk for pregnancy complications, as well as those whose gestational age may have been inaccurate. The exclusion criteria were as follows:

1. Non-Chinese ethnicity.
2. Women with no fetal crown–rump length (CRL) measurements between 5 weeks and 0 days and 15 weeks and 6 days and in whom the difference in gestational age based on the last menstrual period and the fetal crown–rump length (CRL) as measured via ultrasound between 5 weeks and 0 days and 15 weeks and 6 days after the LMP was 7 days or more, using the formula described by Robinson and Fleming[4].

GA (days) = 8.052(CRL×1.037)^1/2^+23.73

1. Women with twin and multiple gestation.
2. Women with disorders that may affect fetal growth: pre-gestational diabetes mellitus and cardiovascular disease (pre-existing hypertension, [heart](http://dict.youdao.com/w/heart/) [failure](http://dict.youdao.com/w/failure/), coronary heart disease, arrhythmia, valvular heart disease).
3. Women with severe pregnancy complications: preeclampsia, eclampsia, HELLP syndrome.
4. Women with abnormal fetal outcomes: fetal malformations (congenital malformations diagnosed by ultrasound during pregnancy or at birth by clinical examination), fetal chromosomal abnormalities, evidence of fetal viral infection (cytomegalovirus infection), fetal death and stillbirth.

**Method of dating pregnancy**

Gestational age was calculated precisely to the day.

**Choosing the reference curve:**

C. Ioannou et al[5] summarized and evaluated 83 studies of fetal biometry before 2012. This study provided basic insight into the ultrasound size charts that had been developed according to different populations worldwide, as well as their quality based on the study design, statistical analysis and reporting methods. In this systematic review, three publications from China were included: *Lei H et al*[6] did not provide the equation for the mean and SD and was thus excluded from our study; *Pang MW et al*[7] customized the fetal biometric charts not only according to gestational age but also according to variables such as maternal and pregnancy characteristics, including booking weight and height, age, parity and fetal sex. This study was excluded because the gestational weeks were between 24 and 40 weeks. We included the charts and reference equations reported in the Hong Kong Chinese population study b*y Leung TN, Pang MW et al*[8], whose study design, statistical analysis and reporting methods were of high quality. The reference curve developed by *Chitty LS, Altman DG* et al[9] was also included in our study because of its high quality despite its early publication and narrow population (UK only). In addition, the latter reference is a classic chart that has been widely used.

Lastly, the reference curve published by the Fetal Growth Longitudinal Study of the INTERGROWTH-21st Project [1], a multi-center, population-based longitudinal study (that included a center in China), was included and evaluated for its suitability. This reference is the latest and newest study evaluating fetal charts that employed a scientific design, strict quality control and rigorous statistical analysis and has garnered widespread attention since its publication.

**Statistical analysis**

FL measurements from 20,089 ultrasound scans from 7,330 patients between 11 and 43 weeks gestation were analyzed. The FL data were then transformed into Z-scores, calculated using three series of reference equations described in three studies: Leung TN, Pang MW et al (2008)[8]; Chitty LS, Altman DG et al (1994)[9]; and Papageorghiou, AT et al (2014)[1].

Statistical analysis was performed using SPSS version 18.0. Z-scores were calculated according to gestational age using the following formula[3]:

***Z-score = (observed FL − expected FL mean)/SD mean.***

The observed FL is the value obtained from the measurements, the expected FL mean is the value for our population calculated from the reference equations at this gestational age, and the SD mean is the SD associated with the mean value calculated from the reference equations at the same gestational age from our population[3].

According to the definition[3], Z-scores should follow a non-skewed standard normal distribution with a mean of 0 and an SD of 1 if the measurements are consistent with the reference equations used to calculate them. By definition[3], in a standard normal distribution, the -1 SD to +1 SD interval includes 68% of the population and the +2 SD interval includes 95% of the population, with the 5^th^ percentile corresponding to -1.645 SD and the 95^th^ percentile corresponding to +1.645 SD.

It is [worth](http://dict.youdao.com/w/worth/) [noting](http://dict.youdao.com/w/noting/) [that](http://dict.youdao.com/w/that/) the applied ranges of gestational weeks for the three reference equations were different. Before analysis, the FL measured outside the application was removed as long as the measurement was more than 5 SDs (because these were regarded as implausible on the basis of gestational age distribution from all of the sites[1])

Each Z-score distribution was expressed as the mean and SD, as well as skewness and kurtosis, which were compared with the standard normal distribution using the Kolmogorov-Smirnov test. The histogram of their distributions was superimposed on the non-skewed standard normal curve (mean=0, SD=1) to provide a direct visual impression.

Finally, the sensitivity and specificity of each reference chart for identifying fetuses <5^th^ or >95^th^ percentile (based on the observed distribution of Z-scores) were then calculated. The Youden index (YI=sensitivity + specificity -1) was also listed. A [scatter](http://dict.youdao.com/w/scatter/) [diagram](http://dict.youdao.com/w/diagram/) with the 5^th^, 50^th^, and 95^th^ percentile curves calculated from each reference chart that was superimposed on it was presented to provide a visual impression.

**Results**

**Baseline demographic characteristics**

Table 2 shows the baseline demographic characteristics for the enrolled population of our study. The median age of the mothers was 28.7 years, and was 31.8 for the fathers. The average maternal and paternal weight before pregnancy was 56.8 kg and 75.7kg. The mean maternal birthweight ± SD was 3233.1±516.6 g. The mean maternal and paternal height ± SD were 162.4±4.8 and 174.9±5.1 cm. The median gestational age of delivery was 39.5 (range, 28–42) weeks. Six thousand three hundred and ninety-seven subjects (87.2%) were nulliparous. Seven thousand and fifty-four (96.2%) delivered at term, two hundred and sixty (3.6%) delivered preterm (< 37 weeks) and sixteen (0.2%) delivered postterm (≥ 42 weeks). The mean birth weight ± SD was 3374.8± 431.4 g.

**Table 2: The demographic characteristics of pregnant women enrolled in this study..**

| **Table 2 Baseline characteristics** | Mean | SD |
| --- | --- | --- |
| Maternal age, years | 28.7 | 3.9 |
| Gestational age, weeks | 39.5 | 1.4 |
| Maternal weight before pregnancy, kg | 56.8 | 8.8 |
| Maternal height, cm | 162.4 | 4.8 |
| Paternal age, years | 31.4 | 4.8 |
| Paternal height, cm | 174.9 | 5.1 |
| Paternal weight before pregnancy, kg | 75.7 | 12.5 |
| Paternal body-mass index, kg/m^2^ | 24.7 | 3.6 |
| Maternal body-mass index before pregnancy, kg/m^2^ | 21.5 | 3.4 |
| Maternal birthweight, g | 3233.1 | 516.6 |
| Age of marriage, year | 26.5 | 3.0 |
| Weight of new-bore, g | 3374.8 | 431.4 |

**Do they match the standard normal distribution**?

The Z-score distribution curves of the measurements appeared to be normal (Figure 1 (a),(b),(c)), but none of them exactly matched the expected standard normal distribution. Table 3 shows the Z-score distribution, which was expressed as the mean and SD, as well as skewness and kurtosis, and the outcome compared with the standard normal distribution using the Kolmogorov-Smirnov test.

A total of 18,896 measurements between 12 and 40 gestational weeks were transformed into Z-scores using the reference equations from Leung TN, Pang MW et al. The mean value of the Z-score was 0.8624, and the SD was 1.09222. Skewness and kurtosis were -0.055 and 0.540, respectively, both less than 1. However, when divided by the [standard](http://dict.youdao.com/w/standard/) [error](http://dict.youdao.com/w/error/) (SE), the results were -3.05 and 15, both [absolute](http://dict.youdao.com/w/absolute/) [value](http://dict.youdao.com/w/value/)s ＞2, thus refuting the normal distribution hypothesis. The result of the Kolmogorov-Smirnov test also confirmed that the Z-score distribution refuted the normal distribution hypothesis (Z=1.544, P=0.017). In the histogram of Z-score distributions with a centered and superimposed standard normal reference curve (Figure1 (a)), the histogram of Z-scores calculated using the Leung TN, Pang MW et al equations was clearly skewed to the left.

Using the reference equations from Chitty LS, Altman DG et al, a total of 19,951 measurements between 12-42 gestational weeks were transformed into Z-scores. The mean value of the Z-scores was -0.1347, and the SD was 0.87240. Skewness and kurtosis were -0.004 and 0.032, respectively, both less than 1. However, when divided by the [standard](http://dict.youdao.com/w/standard/) [error](http://dict.youdao.com/w/error/) (SE), the results were -0.24 and 25.7, the [absolute](http://dict.youdao.com/w/absolute/) [value](http://dict.youdao.com/w/value/) of the latter was ＞2, thus refuting the normal distribution hypothesis. The result of the Kolmogorov-Smirnov test also confirmed this conclusion (Z=1.971, P=0.005). The histogram of Z-score distributions calculated using this equation (Figure1 (b)) seemed to be narrowed compared with the standard normal reference curve.

Finally, we used the reference equations provided by Papageorghiou, AT et al. A total of 19,878 measurements between 14-42 gestational weeks were transformed into Z-scores. The mean value of the Z-scores was 0.5809, and the SD was 1.39380. The skewness and kurtosis were 0.040 and 0.032, respectively, both less than 1. However, when divided by the [standard](http://dict.youdao.com/w/standard/) [error](http://dict.youdao.com/w/error/) (SE), the results were 2.4 and 0.91, and the [absolute](http://dict.youdao.com/w/absolute/) [value](http://dict.youdao.com/w/value/) of the former was ＞2, thus refuting the normal distribution hypothesis. The result of the Kolmogorov-Smirnov test confirmed the same hypothesis (Z=1.721, P=0.007). In the histogram of Z-score distributions with a centered and superimposed standard normal reference curve (Figure1(c)), the histogram of Z-scores calculated using the equations from Papageorghiou, AT et al (2014) seemed to be slightly wider and lower.

**Are they effective at identifying measurements ＜5^th^ or ＞95^th^ percentile?**

From the scatter diagram of the 5^th^, 50^th^, and 95^th^ percentile curves calculated from each superimposed reference chart (Figure 2 (a),(b),(c)), we were able to obtain a rough direct impression. The overall results for the classification of the fetuses using the 5^th^ and 95^th^ percentiles from each of the three reference curves for each parameter are shown in Tables 4 and 5 (see Table 4 and Table 5).

When using the reference equations from Leung TN, Pang MW et al, the observed Z-scores for the 5^th^ percentile and 95^th^ percentile were -0.949 and 2.6627. A total of 715 measurements that were actually less than the 5^th^ percentile were missed diagnoses, and 3,527 measurements were wrongly classified as larger than the 95^th^ percentile. The sensitivity of screening fetuses with measurements < 5^th^ percentile was only 23.4%, although the specificity was 100%, and the Youden index was 0.234. Thus, the value was too low to be used as a diagnostic test. The sensitivity and specificity of screening for fetuses with measurements ＞95^th^ percentile were 100% and 80.3%, respectively, and the Youden index was 0.803.

The observed Z-scores for the 5^th^ percentile and 95^th^ percentile when using the reference equations from Chitty LS, Altman DG et al were -1.532 and 1.2731. A total of 256 measurements that were actually less than the 5^th^ percentile and 604 measurements that were actually larger than the 5^th^ percentile were missed diagnoses. The sensitivity of screening fetuses with measurements < 5^th^ percentile and ＞ 95^th^ percentile was 74.3% and 39.4%, respectively, and the specificity was 100% for both percentiles. Thus, the Youden index was 0.743 for identifying measurements that were ＜5^th^ percentile and 0.394 for identifying measurements that were ＞95^th^ percentile.

Finally, when using the reference equations from Papageorghiou, AT et al (2014), the observed Z-score for the 5^th^ and 95^th^ percentile were -1.6664 and 2.9181. Only 33 measurements were wrongly classified as less than the 5^th^ percentile, and 3,437 measurements were wrongly classified as greater than the 95^th^ percentile. The sensitivity of screening fetuses with measurements < 5th percentile and ＞ 95th percentile were both 100%, and the specificity was 99.8% and 81.6%., respectively. Thus, the Youden index was 0.998 for identifying measurements that were ＜5^th^ percentile and 0.816 for identifying measurements that were ＞95^th^ percentile.

**Discussion:**

Because of ethnic heterogeneity, we were initially inclined to believe that reference curves calculated from data from our own nation and ethnic population would be more applicable. Surprisingly, our findings were very different. The Z-score distribution curves of the measurements appeared to be normal, but none of them matched the expected standard normal distribution. The only published reference curve from a Chinese population (Leung TN, Pang MW et al) had very limited diagnostic value in correctly identifying fetuses with short femur length, with a Youden index of only 0.234. In the clinical arena, however, the significance of correctly diagnosing a fetus with a short femur length is much greater than that of identifying a longer FL. Thus, this curve is not the appropriate reference chart for our Chinese population. This may be due to the fact that this curve was calculated from data from one Hong Kong hospital and that hereditary differences may exist between women in that region and women in northern China.

The classic reference curve published by Chitty LS, Altman DG et al in 1994 has been widely used. The Youden index for recognizing a FL＜ the 5^th^ percentile is 0.743. A Youden index of more than 0.7 is generally considered to be a better diagnostic test. Although its diagnostic value for recognizing a FL ＞5^th^ percentile is lower (Youden index 0.394), we still considered this reference curve to be valuable in the clinical arena.

Papageorghiou, AT et al published the study of the Fetal Growth Longitudinal Study of the INTERGROWTH-21st Project. This was a multi-center, population-based longitudinal study that aroused widespread concern in the obstetrics academic community globally because of its scientific design. Doubts still remain. The data were collected from 8 countries, and we were uncertain about the applicability of the reference curve in our population. We were surprised to discover that this chart had very high sensitivity and specificity for correctly identifying measurements below the 5^th^ percentile and measurements greater than the 95^th^ percentile, with Youden indices of 0.998 and 0.816, respectively. Thus, this reference chart was found to have the best diagnostic value in our study.

**Limitations:**

Our study evaluated different reference standards for measuring femoral length (FL) in a Chinese population. However, it has some limitations.

Recent studies about customized percentiles that are adjusted or customized based on sex and maternal characteristics, such as height, weight, parity, and ethnic origin, have conveyed the idea that one size does not fit all[10]. Instead of customized percentiles, our study used population percentiles for the following reasons: first, it was not permitted to report fetal sex on ultrasound because of Chinese government policies, and fetal sex and weight are two of the most important factors in customized percentiles. Therefore, despite its advantages, the clinical use of customized growth charts is limited in in terms of retrospective value and clinical decision making in China. Second, our data were collected in 2013, when China was enforcing the one-child policy, which meant that most families had only one child (11,985 of 15,194 women were primiparous in our study). Taking these unique national policies into consideration, we still used the traditional population percentiles in our study. We attempted to address this limitation by setting exclusion criteria designed to reduce individual differences, for example, by excluding women who were not ethnically Chinese, had twin or multiple pregnancies, had disorders that may affect fetal growth and had severe pregnancy complications.

This policy may change some day when people no longer prefer boys to girls. We thus expect that the customized growth charts will present a real benefit to mothers.

**Conclusions:**

It is important to choose an appropriate chart when defining what is normal. The Papageorghiou reference curve for FL seems to best fit our population It may be time to change our reference curve for femur length.
